# Magnetic Resonance Imaging Revealed Splenic Targeting of Canine Parvovirus Capsid Protein VP2

**DOI:** 10.1038/srep23392

**Published:** 2016-03-21

**Authors:** Yufei Ma, Haiming Wang, Dan Yan, Yanquan Wei, Yuhua Cao, Peiwei Yi, Hailu Zhang, Zongwu Deng, Jianwu Dai, Xiangtao Liu, Jianxun Luo, Zhijun Zhang, Shiqi Sun, Huichen Guo

**Affiliations:** 1 State Key Laboratory of Veterinary Etiological Biology, Lanzhou Veterinary Research Institute, Chinese Academy of Agricultural Sciences, Xujiaping No1, Lanzhou, Gansu, 730046, China; 2 CAS Key Laboratory of Nano-Bio Interface, Division of Nanobiomedicine, CAS Center for Excellence in Nanoscience, Suzhou Institute of Nano-Tech and Nano-Bionics, Chinese Academy of Sciences, Suzhou 215123, China

## Abstract

Canine parvovirus (CPV) is a highly contagious infectious virus, whose infectious mechanism remains unclear because of acute gastroenteritis and the lack of an efficient tool to visualize the virus in real time during virology research. In this study, we developed an iron oxide nanoparticle supported by graphene quantum dots (GQD), namely, FeGQD. In this composite material, GQD acts as a stabilizer; thus, vacancies are retained on the surface for further physical adsorption of the CPV VP2 protein. The FeGQD@VP2 nanocomposite product showed largely enhanced colloidal stability in comparison with bare FeGQD, as well as negligible toxicity both *in vitro* and *in vivo*. The composite displayed high uptake into transferrin receptor (TfR) positive cells, which are distinguishable from FeGQD or TfR negative cells. In addition, the composite developed a significant accumulation in spleen rather than in liver, where bare FeGQD or most iron oxide nanoparticles gather. As these evident targeting abilities of FeGQD@VP2 strongly suggested, the biological activity of CPV VP2 was retained in our study, and its biological functions might correspond to CPV when the rare splenic targeting ability is considered. This approach can be applied to numerous other biomedical studies that require a simple yet efficient approach to track proteins *in vivo* while retaining biological function and may facilitate virus-related research.

Canine parvovirus (CPV), a member of the *Parvovirus* genus, is an important pathogen that mostly affects dogs. Since its emergence in 1977, CPV has developed several mutants that are widely distributed across the world^1^. Given the highly contagious nature of this infectious virus, infected dogs usually develop acute gastroenteritis in three to seven days, with symptoms of appetite loss, vomiting, fever, diarrhea, and leucopenia^2^. At present, vaccination seems to be the most effective and efficient method of controlling the spread of CPV infection of dogs. However, vaccinated dogs can still die from CPV infections, and worse, unvaccinated dogs may act as virus carriers without showing any symptoms and affect other susceptible dogs^3^. Therefore, understanding the infectious mechanism of CPV is an urgent concern. Such a study will depend on the development of an efficient approach to facilitate virology research during virus characterization and *in vivo* tracking.

Typically, parvovirus presents a spherical capsid 25–30 nm in diameter and constructed with three proteins and a single-strand DNA molecule^2^. According to X-ray crystallography, a parvovirus capsid contains 60 copies of three proteins, VP1, VP2, and sometimes VP3. As VP2 is the main structure protein of CPV, accounting for ~90% of all the capsid proteins and representing a major part of VP1, it represents the dominant determinant of host range and virus–host interactions. CPV VP2 has been expressed and produced to build virus-like particles (VLPs)^4^,^5^. These VLPs stimulate both cellular and humoral immune responses as a candidate vaccine for the prevention of CPV-associated diseases^6^,^7^. Despite certain successful applications of CPV VLPs in previous reports^4^,^5^,^6^,^7^,^8^, whether CPV VP2 or VLPs maintain the tissue tropism of the native virus *in vivo* remains unclear.

Magnetic resonance imaging (MRI) represents a modern and non-invasive approach for disease diagnosis and *in vivo* biomedical studies. Normally, MRI tests require specific MRI agents, such as gadolinium complexes^9^ and superparamagnetic iron oxide nanoparticles (SPION)^10^,^11^. For SPION, numerous efforts have been focused on exploring their biomedical applications and applying them to clinical diagnosis^10^,^11^,^12^. However, biomedical scientists are not satisfied with the current synthetic routes developed for water-dispersible SPION mainly because of the low adaptability and poor affinity of SPION for binding functional proteins. Thermal decomposition, a widely used and reliable method for the preparation of SPION, produces mono-dispersed nanoparticles but requires further modification to enable biomedical purposes. Co-precipitation and hydrothermal approach can directly provide water-dispersible products but normally comes with large degrees of polydispersity and particle aggregation. Most of all, to reduce Fe (III) and stabilize the obtained nanoparticles, these methods require organic molecules that occupy the surfaces of SPION and consequently prevent the attachment of desired biomolecules. As a result of the exhausted process for the conjugation of specific biologic ligands, research on the biomedical applications of SPION has recently become routine and unproductive.

According to our previous study^13^, graphene quantum dots (GQD) can serve as stabilizers and constrain the size of gold nanoparticles that are *in situ* grown on the GQD surface. As no organic additives are introduced into the system, the gold nanoparticles possess abundant vacancies and accommodate hundreds of proteins for each particle. This approach is expected to be suitable for the preparation of other metallic nanoparticles. Therefore, in this work, SPION are grown *in situ* on the GQD surface to obtain nanoparticles with “clean surfaces” for the further functionalization of specific proteins. By combining with CPV VP2 attached onto this SPION, we will assess the bioactivity of VP2 or VLPs and study if VP2 maintains the targeting ability (or tissue tropism) of CPV with the help of MRI, which visualizes SPION *in vivo* (Fig. 1). Through verifying the targeting ability of VP2 generated from CPV, the current study can facilitate virus-related research and accelerate the biomedical applications of metallic nanoparticles for specific purposes.

## Results and Discussion

### CPV VP2 proteins

CPV VP2 protein was expressed in *Escherichia coli* and purified by nickel affinity chromatography, as previously reported^6^. VP2 proteins were subsequently analyzed by SDS–PAGE (Fig. 2A) and Western blot (Fig. 2B). Consistent with previous reports^6^, our results showed that the His–SUMO–VP2 fusion protein is approximately 87 kDa and the VP2 protein cleaved with the SUMO protease is approximately 65 kDa.

Maintaining the bioactivity of VP2 protein is an important consideration in our study. For simple verification of the biological activity of VP2 proteins expressed in *E. coli*, VP2 was self-assembled into CPV VLPs. The transmission electron microscopy (TEM) image (Fig. 2C) showed that the VP2 assembled VLPs displayed a homogeneous particle size of ~25 nm and a shell thickness of 3 nm, which are comparable to the size of the VP2 protein. In the UV–vis spectra (Fig. 2D), a 6 nm blue shift of the emission wavelength in comparison with VLPs with VP2 protein was observed. This phenomenon is caused by the aromatic protein residues, including tryptophan (Trp), phenylalanine (Phe), and tyrosine (Tyr), which can generate intrinsic fluorescence and provide important information on the protein structural change process. The Trp residues of CPV VLPs were merged in the protein structure, resulting in a blue fluorescence peak shift from 316 nm to 310 nm^14^. The successful assembly of VP2 into VLPs confirms that the VP2 proteins expressed in bacteria retains their natural bioactivity, ensuring the success of subsequent modification of FeGQD by VP2 protein.

### FeGQD nanoparticles

GQD were obtained through oxidative cutting of graphite flakes in H_2_SO_4_/HNO_3_ mixture, as described in our previous report^15^. A precipitation method was then applied to allow the *in situ* growth of iron oxide nanoparticles on GQD surface and finally generate the FeGQD nanocomposites. Without GQD, the precipitation of iron ions generated large and aggregated nanoparticles (Fig. 3A). In contrast, the addition of GQD to the same recipe resulted in ultra-small nanoparticles, FeGQD, 3–5 nm in size (Fig. 3B), which is comparable to the size of bare GQD. These results validate the supporting function of GQD flakes. The AFM image (Fig. 3C) also displays a larger height for FeGQD (~5 nm) than for GQD (0.5–1 nm), suggesting the successful deposition of iron oxide nanoparticles on GQD.

### FeGQD@VP2 nanocomposites

For specific targeting, FeGQD nanoparticles were coated with CPV VP2 proteins through simple adsorption, which was accomplished by mixing FeGQD with VP2 and stirring overnight. Unattached VP2 proteins were separated through high-speed centrifugation because FeGQD@VP2 forms larger nanoparticles than VP2 protein. The FeGQD@VP2 complex was characterized by UV–vis spectra (Fig. 4A). Absorbance showed an identical absorbance peak of VP2 for FeGQD@VP2; this peak resembled the absorbance of FeGQD throughout the UV–vis region. This finding suggests that VP2 proteins were successfully coated onto the surface of FeGQD nanoparticles. In addition, based on absorption at 280 nm, it is roughly estimated that a FeGQD nanoparticle (100−500 kDa according to the particle size) was coated with approximately 10–50 VP2 proteins.

As shown in Fig. 4B, FeGQD@VP2 displayed a deep blue color on the gel after staining with Coomassie blue R250, indicating the presence of VP2 in the nanocomposites. In comparison, FeGQD nanoparticles only exhibited a light blue color after further Prussian blue staining. FeGQD@VP2 composites also ran slower than FeGQD in the gel, suggesting the binding of VP2 proteins to FeGQD nanoparticles.

**Figure 1:**
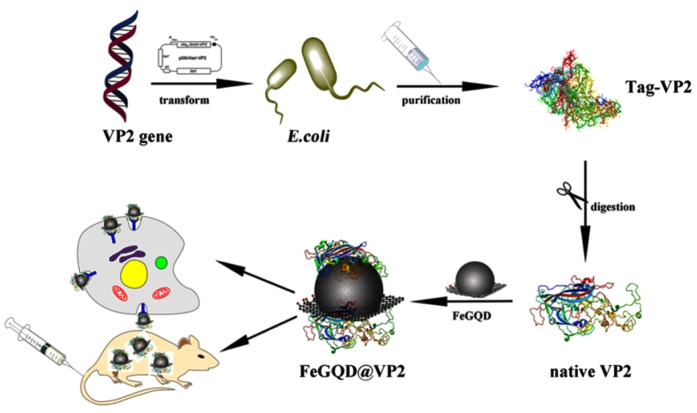
Schematic showing the preparation of FeGQD nanoparticles coated with canine parvovirus VP2 protein for MR imaging *in vitro* and *in vivo*.

According to TEM images, the morphological property of FeGQD@VP2 compared with bare FeGQD clearly suggests the interaction between FeGQD and VP2. FeGQD@VP2 shows 10–50 nm clusters of FeGQD assembled with the help of VP2, in association with several VLPs surrounded by FeGQD aggregates (Fig. 4C–E). Owing to the surface-coating VP2 proteins, FeGQD@VP2 nanocomposites showed superior stability in ionic solutions and cell culture media compared with bare FeGQD (Supplemental information Fig. 1). Notably, FeGQD was also stable in fetal bovine serum (FBS), clearly suggesting that FeGQD adsorbs various proteins because no organic ligands were added as reducers or stabilizers during its synthesis.

In addition, the hydrodynamic sizes of FeGQD and FeGQD@VP2 (in phosphate-buffered saline (PBS, pH 7.4) are 20.0 ± 3.7 and 43.1 ± 4.6 nm (Table 1) respectively. This finding indicates the assembly of FeGQD through VP2 binding and the excellent dispersity of these nanocomposites in aqueous solutions.

### T2 reflexivity measurement of FeGQD@VP2

Iron oxide-based particles can create strong local magnetic field gradients that may be reflected by the rapid loss of signals in *T*_*2*_-weighted images. To investigate the magnetic property of FeGQD@VP2, phantom experiments were conducted using MRI and a series of Fe concentrations. As shown in the *T*_*2*_-weighted MRI images (Fig. 5), both FeGQD and FeGQD@VP2 can induce a decrease in MR signal intensity upon increased Fe concentration. The increased transverse relaxivities (*r*_*2*_) of FeGQD@VP2 and FeGQD are 134.3 and 126.6 mM^−1^s^−1^ respectively. The *r*_*2*_ value of FeGQD@VP2 is higher than that of FeGQD, a discrepancy that may be ascribed to the clusters of Fe-containing nanoparticles^16^,^17^. Considering the excellent stability of FeGQD@VP2 in cell culture media, the nanoparticles can be safely employed as sensitive MRI contrast agents for *in vivo* tracking of VP2 proteins.

### Toxicity of FeGQD@VP2

**Figure 2:**
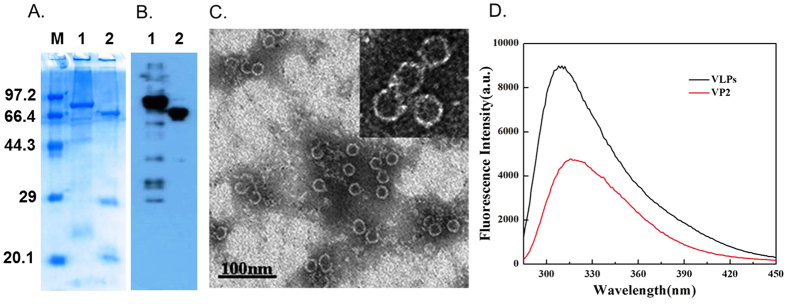
Purification and characterization of CPV VP2 proteins and VLPs. (**A**) SDS–PAGE of His–SUMO–VP2 proteins cleaved with enzyme (Lane 2, VP2 protein) or without enzyme (Lane 1, His-SUMO-VP2). (**B**) Western blot of His–SUMO–VP2 proteins cleaved with enzyme (Lane 2, VP2 protein) or without enzyme (Lane 1, His–SUMO–VP2). (**C**) TEM image of CPV VLPs. (**D**) Fluorescence spectra of VP2 and VLPs in PBS solution.

**Figure 3:**
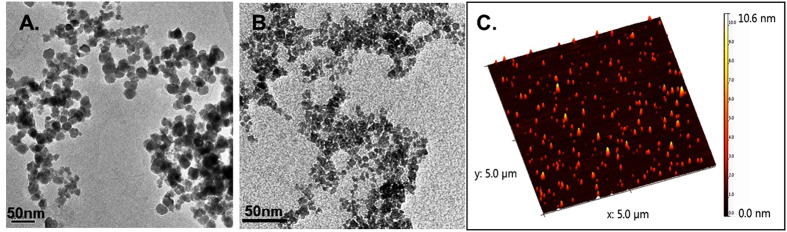
TEM image of SPION. Co-precipitation method shows the larger size and aggregation of GQD particles (**A**); FeGQD shows considerably smaller size and good size homogeneity in the TEM (**B**) and AFM (**C**) images.

**Figure 4:**
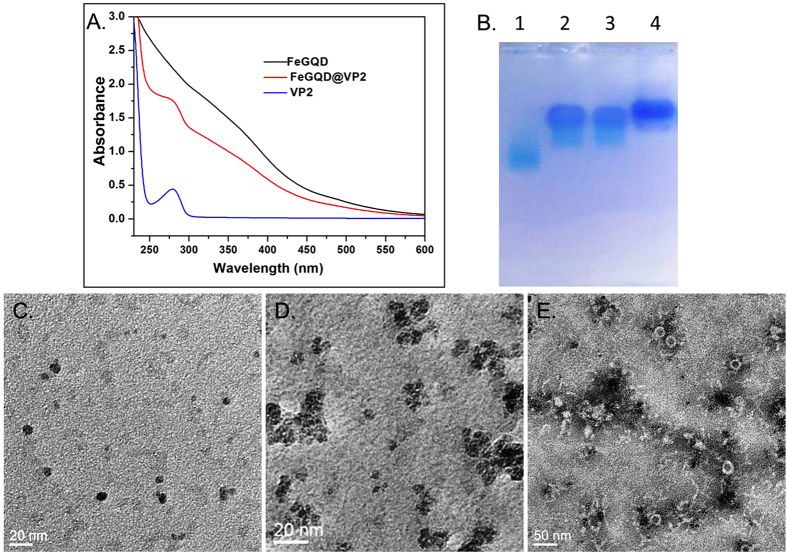
Coating of FeGQD with CPV VP2 proteins. (**A**) UV–vis spectra of VP2 (1 mg mL^−1^), FeGQD (0.5 mg mL^−1^), and FeGQD@VP2 (0.8 mg mL^−1^); (**B**) Agarose gel electrophoresis; the sample was stained with Coomassie blue R250 and Prussian blue staining respectively. Lane 1: FeGQD, Lane 2, and Lane 3: FeGQD@VP2, Lane 4: VP2 protein; (**C**) TEM images of FeGQD; (**D**) TEM images FeGQD@VP2 without phosphotungstic acid staining; (**E**) TEM images FeGQD@VP2 stained by phosphotungstic acid.

**Figure 5:**
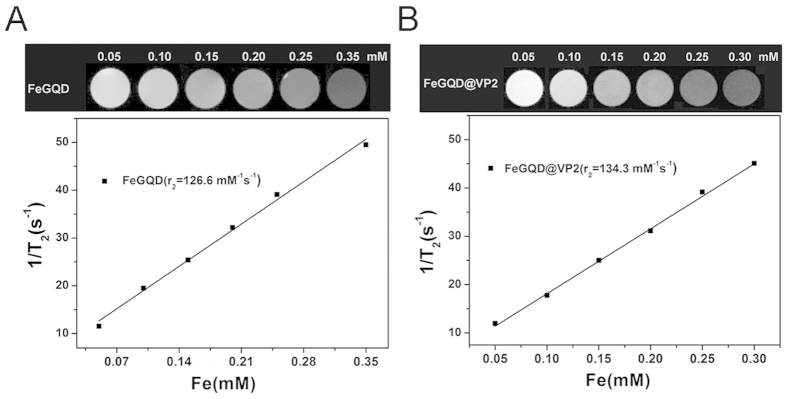
Magnetic properties of FeGQD and FeGQD@VP2. *T*_*2*_ weighted images and linear fitting of 1*/T*_*2*_ of FeGQD (**A**) and FeGQD@VP2 (**B**) with different concentrations in PBS (pH 7.4).

**Figure 6:**
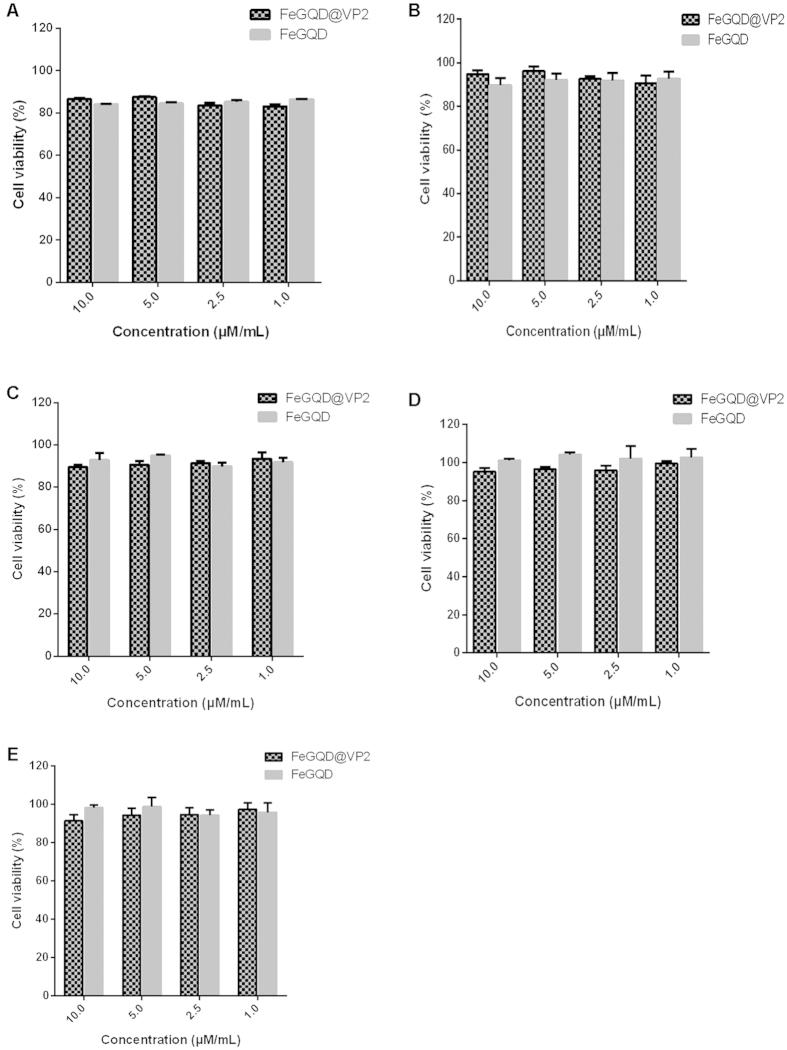
Cytotoxicity of FeGQD@VP2 in different cell lines. MTT assay of relative viability of A549 (**A**), BHK-21 (**B**), F81 (**C**), HeLa (**D**), and MCF-7(**E**) cells incubated with FeGQD@VP2 at different Fe concentrations for 24 h, respectively.

To assess the cytotoxicity of FeGQD@VP2 nanoparticles, MTT assays were performed with different types of cells, including tumor cell lines A549, MCF-7, and Hela, as well as CPV-targeting cell line F81, all of which express transferin receptors. Given its immunity to CPV, the BHK-21 cell was used as a negative control. As shown in Fig. 6, both FeGQD nanoparticles and FeGQD@VP2 nanocomposites did not show significant toxicity to different cell lines. This finding is consistent with the hemocompatibility of FeGQD@VP2 nanoparticles, in which FeGQD@VP2 did not show any evident hemolytic activities in the RBC solution in comparison with the PBS solution as a negative control (Supplemental information Fig. 2). Hence, FeGQD@VP2 nanoparticles may be used with good biocompatibility and low toxicity in numerous cell lines.

### Cellular targeting uptake of FeGQD@VP2 nanoparticles

CPV VP2 proteins perform vital functions in deciding virus host range, cell binding, and entry^18^,^19^. Given that FeGQD@VP2 nanocomposites are composed of a VP2 protein shell, they may remain as the targeting properties of VP2 protein. Different cell lines, such as F81 (transferrin receptor positive), BHK21 (transferrin receptor negative), and HeLa cells as positive control, are used to confirm the targeting ability of FeGQD@VP2 nanoparticles. Prussian blue staining, a useful tool for visualizing the iron content of cells, was used to estimate the cell entry capacity of FeGQD@VP2 nanoparticles.

**Figure 7:**
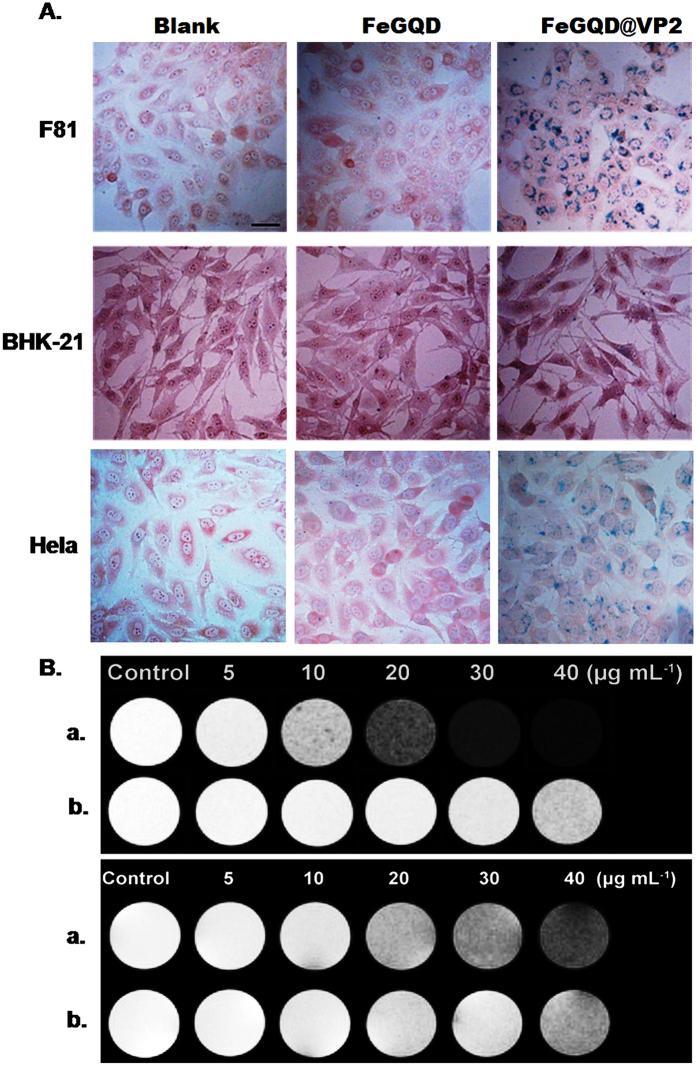
Targeting of nanoparticles in cells. **(A)** Photomicrographs of F81 cells, BHK-21 cells, and Hela cells incubated with FeGQD or FeGQD@VP2 and subsequently stained with Prussian blue. (**B**) *T*_*2*_-weighted MRI of F81 cells (upper) or BHK21 cells (under) after treatment with FeGQD@VP2 (**a**) or FeGQD (**b**) at a series of Fe concentrations (5, 10, 20, 30, and 40 μg mL^−1^) for 24 h.

As Fig. 7A shows, F81 cells incubated with FeGQD@VP2 NPs at 10 μg mL^−1^ Fe for 24 h show a large number of clusters of dense blue iron granules in the cytoplasm. HeLa cells display light-blue clusters, whereas BHK21 cells present several blue spots in the cytoplasm under the same condition. In contrast, all cell lines incubated with FeGQD nanoparticles did not show an evident blue color after Prussian blue staining. These results indicate that F81 and Hela cells exhibit considerably higher cellular uptake than BHK21 cells. The results also suggest that transferrin receptor-positive cells can take up a large amount of FeGQD@VP2 but not FeGQD, thereby confirming the targeting properties of VP2 protein after adsorbing onto the FeGQD surface.

To assess the effectiveness of MR imaging of FeGQD@VP2, we first conducted *in vitro* imaging experiments with F81 and BHK-21 cells. The cells were incubated with FeGQD@VP2 nanoparticles at different Fe concentrations, and MRI images of F81 cells were obtained and compared with those of BHK21 cells as a control. A comparison between the *T*_*2*_ MR signal intensities of F81 (Fig. 7B) and BHK21 (Fig. 7C) cells incubated with different Fe concentrations demonstrated that MR signal intensity decreases with increasing Fe concentration. However, the relaxation time of F81 cells incubated with FeGQD@VP2 nanoparticles was significantly shorter than that of BHK21 cells under the same experimental conditions. This result was obtained mainly because F81 cells enact a substantially higher cellular uptake of FeGQD@VP2 nanoparticles than BHK21 cells, again demonstrating that FeGQD@VP2 possesses specific targeting of the transferrin receptor. This finding corresponds to the cellular results of Prussian blue staining and the targeting properties of VP2.

### *In vivo* MR imaging

**Figure 8:**
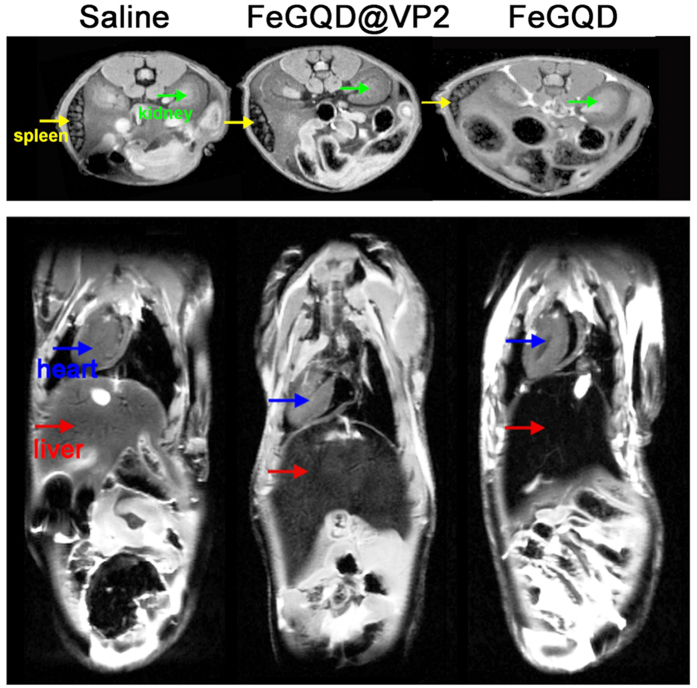
*In vivo* MR imaging. *T*_*2*_ weighted MR images of mice at 24 h post-intravenous injection of saline, FeGQD, and FeGQD@VP2, respectively.

For *in vivo* MR imaging, 5–6-week-old BALB/c mice were intravenously injected with FeGQD or FeGQD@VP2 at a dose of 5 mg kg^−1^ and anaesthetized after 24 h of injection to obtain *T*_*2*_ weighted images on a micro-MRI system. As Fig. 8 shows, the FeGQD nanoparticles display significant accumulation in the liver region, a finding that is consistent with the results from previous reports that nanoparticles are mainly excreted by liver or spleen^20^,^21^,^22^,^23^. However, compared with FeGQD, FeGQD@VP2 nanocomposites presented more evident darkening effects in the spleen by *T*_*2*_ weighted MRI. VP2 protein may retain its targeting property after being conjugated to FeGQD nanoparticles.

### *In vivo* biodistribution

**Figure 9:**
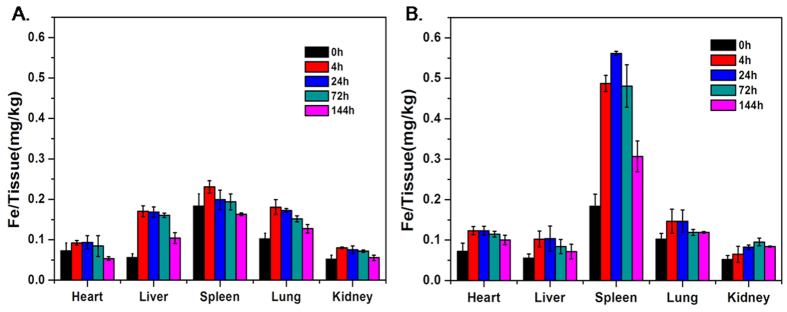
Biodistribution of nanoparticles in mouse organs. FeGQD (**A**) or FeGQD@VP2 (**B**) was injected into mice intravenously with a dosage of 5 mg kg^−1^ body weight. The concentration of Fe in different tissues was detected by ICP–MS at various time points post-injection.

The *in vivo* MRI results showed that the VP2 protein significantly aids FeGQD to target the spleen. To further validate the different tissue tropisms of FeGQD and FeGQD@VP2 nanoparticles, an analysis of the *in vivo* biodistribution of these nanoparticles was conducted. Female BALB/c mice were intravenously injected with 5 mg kg^−1^ of nanoparticles, sacrificed at certain time points for organ collection, and then analyzed for Fe concentration through inductively coupled plasma–atomic emission spectrometry (ICP–AES). As shown in Fig. 9, the nanocomposites were almost spread in all major organs. The FeGQD nanoparticles mainly accumulated and remained in the liver, lungs, and spleen. However, significant concentrations of FeGQD@VP2 were present in spleens compared with the concentrations in other organs. Interestingly, FeGQD@VP2 appeared to aptly locate in the spleen compared with FeGQD. This tendency could be related to the VP2 protein modification and is consistent with the result of MRI *in vivo*.

Nanoparticles usually accumulate in organs, including liver, spleen, and lung. This fact is also demonstrated by the MRI results and the *in vivo* distribution of FeGQD or FeGQD@VP2 nanoparticles in our study. However, the existence of FeGQD@VP2 in spleen is notable. In fact, most iron oxide nanoparticles that are either coated with phospholipids or proteins show significant accumulation in the liver of mice rather than the spleen^24^,^25^. Considering the fact that the FeGQD@VP2 mainly accumulates in the spleen, we suppose that the targeting ability of the nanoparticles is provided by CPV VP2. On the other hand, CPV or CPV VLPs are commonly accepted to possess tropism in the immune system, especially spleen and lymph node, and VP2 is known to dominate the structure proteins and resemble most of the biological properties of CPV^2^,^26^. Therefore, we proposed that our FeGQD@VP2 particles could be considered to reflect the bioactivity of CPV VP2 protein *in vivo* to a certain extent. That is, the utilization of metallic nanoparticles with “clean” surfaces can facilitate virus-related research through the combination of metal particles with key compartments from the virus and visualizing their fate with more accessible techniques.

Although these major organs showed apparent FeGQD accumulation at 24 h, the Fe concentrations also decreased after 144 h. This finding suggests that nanoparticles will be cleared from the animal body and a lack of potential biotoxicity.

## Conclusion

Iron oxide nanoparticles were successfully grown *in situ* onto the surface of GQDs, and then coated with CPV VP2 to enable specific targeting ability and improve their colloidal stability. Owing to the clean surface of GQD, conjugation was simply processed through physical adsorption and may be applicable to other proteins. In addition, the FeGQD nanoparticles formed larger clusters and exhibited slightly enhanced MRI performance. This FeGQD@VP2 complex displayed good *in vitro* and *in vivo* biocompatibility, as well as demonstrated specific targeting of transferrin receptor-positive cells. *in vitro* MRI experiments also exposed the preferred accumulation in certain cells, demonstrating the specific targeting ability of FeGQD@VP2 and its ability to be visualized inside the cells with non-invasive MRI techniques. Further *in vivo* experiments validated the excellent safety of FeGQD@VP2 and the potential to serve as MIRI agents, as well as revealed the splenic targeting ability of the nanocomposites. In comparison with other materials, either iron oxide nanoparticles or virus-like nanoparticles, the rare splenic targeting may resemble the biological activity of CPV. The simple and reliable method can be widely applied to other viral studies and may engender advanced biological studies.

## Materials and Methods

### Materials

Ferrous chloride tetrahydrate (FeCl_2_•4H_2_O, >99%), ammonia (25%–28% NH_3_ in water solution), and dimethyl sulfoxide (DMSO) were purchased from Aladdin Reagent Corporation. Ni–NTA Sefinose^TM^ resin and a small chromatographic column were obtained from Shanghai Sangon Biological Engineering Technology and Services Corporation. Enhanced BCA protein and MTT assay kits were purchased from Beyotime Biotechnology Corporation. Imidazole and TritonX-100 were supplied by Amresco Corporation. Dulbecco’s modified Eagle’s medium, RPMI 1640 culture medium, and trypsin were acquired from Corning Corporation. McCOY’s 5A, FBS, penicillin, and streptomycin were obtained from Gibco Corporation. Various cell types were obtained from the Institute of Biochemistry and Cell Biology of the Chinese Academy of Sciences. All other chemicals and solvents used were obtained from Sinopharm Chemical Reagent Corporation.

### Synthesis of FeGQD nanoparticles

GQD was synthesized as previously reported^15^. For the preparation of FeGQD, 3 mg GQD in 30 mL water was heated to 80 °C to remove dissolved O_2_, and then 15 mg FeCl_2_•4H_2_O and 0.5 mL 10% NH_3_•4H_2_O were added to the solution. The mixture was stirred at 80 °C for 4 h, cooled to room temperature, and dialyzed (8–14 k MWCO) against water for 12 h. The final solution was collected and stored at 4 °C.

### Characterization of FeGQD nanoparticles

For TEM analysis of FeGQD, 20 μL of the FeGQD samples diluted in deionized water was dropped onto a carbon-coated copper grid and air-dried overnight at room temperature. The sample was observed by TEM (FEI Tecnai G2 F20 S-Twin transmission electron microscope). For atomic force microscopy (AFM) analysis, 50 μL of the samples diluted in deionized water was dropped onto a silicon slice and air-dried at room temperature. The sample was measured by a Vecco Dimension 3100 atomic force microscope. The hydrodynamic sizes and size distributions of FeGQD were measured by using a Malvern Nanosizer (Malvern; Zetasizer Nano ZS90).

### Expression and purification of CPV VP2 protein

The expression and purification of CPV VP2 proteins were performed as described previously^6^. In brief, the VP2 open reading frame was inserted into the pSMK vector to generate the plasmid pSMK-VP2, which was coded for a 6× His tag and a SUMO tag located at the N terminus of VP2 gene. The recombinant plasmid pSMK-VP2 was transformed into *E. coli* RIL, which was then grown in an LB medium containing 50 μg mL^−1^ kanamycin and 34 μg mL^−1^ chloramphenicol at 37 °C and induced with isopropyl-*β*-D-thiogalactoside at 16 °C for 16 h. Cells were harvested by centrifugation and washed once by buffer A (20 mM *Tris*-HCl, 500 mM NaCl, 20 mM imidazole, 2 mM DTT, and 0.05% Triton X-100, pH = 8.0). The cells were then sonicated in buffer A. The supernatant was transferred to a nickel affinity chromatography resin column pre-equilibrated with buffer A, and the column was washed with buffer A. Finally, the protein was eluted with buffer B (20 mM *Tris*-HCl, 500 mM NaCl, 500 mM imidazole, 2 mM DTT, and 5% glycerol, pH = 8.0). The purified His–SUMO–VP2 protein and SUMO enzyme were mixed and dialyzed against buffer C (20 mM *Tris*-HCl, 150 mM NaCl, 5 mM DTT, and 5% glycerol, pH = 7.4) at 4 °C for 20 h. Afterward, the mixture was layered on top of 10%–60% sucrose gradients and centrifuged at 38,000 rpm and 4 °C for 4.5 h. The protein content in each 0.5 mL fraction was roughly determined by SDS–PAGE. Sucrose was removed by diluting in 1000× PBS buffer and ultra-filtering with an Amicon Ultra-15 Centrifugal Filter Unit-30,000NMWL. TEM and fluorescence spectral (Hitachi F4600 fluorescence spectrophotometer) results confirmed that VP2 proteins can assemble into VLPs. The concentration of VP2 was determined by a Bio-Rad protein assay kit.

### Preparation of VP2-coated FeGQD

Exactly 1 mL of VP2 protein (2 mg mL^−1^) was added to 4 mL of 0.5 mg mL^−1^ FeGQD solution. The mixture was incubated at 4 °C for 24 h with stirring and collected by centrifuging at 8000 rpm for 15 min. The pellet was washed twice with pure water and dispersed in pure water once more. The final FeGQD@VP2 complex was detected by SDS–PAGE, UV–vis (Shimadzu UV-2550 spectrophotometer), and TEM.

### MRI

MRI was performed by an 11.7 T Bruker Micro 2.5 micro-MRI system. For the MRI of FeGQD, FeGQD concentration was determined by ICP–OES and diluted to 0.05, 0.10, 0.15, 0.20, 0.25, and 0.30 mM. All samples were transferred to microtubes for MRI with the following parameters: repetition time (TR) = 5000 ms, echo time (TE) = 40 ms, imaging matrix = 128 × 128, slice thickness = 0.6 mm, and field of vision (FOV) = 1.60 cm × 1.60 cm.

For the MRI of F81 and BHK21 cells, the cells were seeded in six-well plates at a density of 3 × 10^6^ cells/well and incubated for 18 h at 37 °C in an atmosphere with 5% CO_2_. FeGQD or FeGQD@VP2 with a Fe concentration of 0, 5, 10, 20, 30, or 40 μg mL^−1^ was added to the cells and incubated at 37 °C for 24 h. The cells were then mixed with 1% low-melting agarose (v/v = 1:1) at a certain cell density, transferred into an NMR tube, and detected by the MRI system. *T*_*2*_ weighted images were obtained by using the multi-slice multi-echo protocol on the micro-MRI system with the following parameters: TR = 5000 ms, TE = 40 ms, imaging matrix = 128×128, slice thickness = 0.6 mm, and FOV = 1.60 cm × 1.60 cm.

For MRI *in vivo*, all experiments were carried out according to the protocols approved by the Institutional Committee for Animal Care and the policy of the National Ministry of Health. 5–6-week-old BALB/c female mice were randomly divided into three groups (3 animals each). FeGQD or FeGQD@VP2 was intravenously injected into mice at a dose of 5 mg kg^−1^. MRI was conducted on the micro-MRI system by conventional spin-echo acquisition. *T*_*2*_ weighted images were obtained 24 h after injection by using the following parameters: TR = 2500 ms, TE = 11 ms, slice thickness = 0.8 mm, imaging matrix = 128 × 128/256 × 128, and FOV = 2.5 cm × 2.5 cm/5 cm × 2.5 cm.

### Cellular viability assay

To evaluate the cell viability, we conducted an MTT assay of the A549, BHK-21, F81, HeLa, and MCF-7 cell lines after treatment with FeGQD or FeGQD@VP2. In brief, the cells were seeded into 96-well plates at initial densities of 1 × 10^4^ cells per well in 200 μL of cell culture medium. After incubation at 37 °C for 18 h, 200 μL of fresh medium with FeGQD or FeGQD@VP2 and different Fe concentrations was added into 96-well plates and incubated at 37 °C for 24 or 48 h. A 20 μL volume of MTT reagent was added to each well, and the plate was incubated further for 4 h. The culture medium in each well was discarded and replaced with 150 μL of DMSO to dissolve the insoluble formazan crystals. The 49 nm absorbance was measured by a multi-label reader (Perkin Elmer Victor X4 multi-label reader).

### Prussian blue staining

F81 and BHK21 cells were seeded into 24-well plates at a density of 6 × 10^4^ cells/well and incubated for 18 h. FeGQD and FeGQD@VP2 were added at the indicated concentration, and the cells were incubated at 37 °C for 24 h. The cells were then washed with a PBS buffer to remove dissociated nanoparticles and fixed with 4% paraformaldehyde at room temperature for 30 min. After washing with PBS, the cells were incubated with Pearls’ reagent (2% potassium ferrocyanide and 2% HCl, v/v = 1:1) for 30 min at room temperature and counterstained with 0.3% neutral red for another 3 min. Finally, the cells were washed with PBS and detected by an upright metallurgical microscope (Leica DM4000M).

### Biodistribution of FeGQD or FeGQD@VP2 in mice

BALB/c female mice were intravenously administered with FeGQD or FeGQD@VP2 at dose of 5 mg kg^−1^. Saline was intravenously injected into mice as a control. The heart, liver, spleen, lung, and kidney of the mice were extracted at different time points after injection, and the organs were cut into 1 mm^3^ pieces and weighed. Tissue samples were placed in aqua regia solution (nitric acid/hydrochloric acid, v/v = 1:3) and digested for 2 d. Fe concentrations in different organs were finally measured by ICP–AES (ICP spectrometer, iCAP 6300 series, Thermo).

## Additional Information

**How to cite this article**: Ma, Y. *et al.* Magnetic Resonance Imaging Revealed Splenic Targeting of Canine Parvovirus Capsid Protein VP2. *Sci. Rep.* **6**, 23392; doi: 10.1038/srep23392 (2016).

## Supplementary Material

Supplementary Information

## Figures and Tables

**Table 1:** Hydrodynamic size and zeta potential of FeGQD and FeGQD@VP2.

Sample	Zeta potential (m V)	Hydrodynamic size (n m)
FeGQD	−36.8 ± 5.82	20.04 ± 3.71
FeGQD@VP2	−27.6 ± 6.51	43.12 ± 4.66
