# Supplementary material for: Magnetic Resonance Imaging Revealed Splenic Targeting of Canine Parvovirus Capsid Protein VP2
Source: Sci Rep. 2016 Mar 21;6:23392. doi: 10.1038/srep23392 (PMC4800397; doi:10.1038/srep23392)
Supplement: Supplementary Information [file srep23392-s1.pdf]

Magnetic Resonance Imaging Revealed Splenic Targeting of Canine Parvovirus Capsid Protein VP2

Yufei Ma[a,b‡], Haiming Wang[a,b‡], Dan Yan[a], Yanquan Wei[a], Yuhua Cao[b], Peiwei Yi[b], Hailu Zhang[b], Zongwu Deng[b], Jianwu Dai[b], Xiangtao Liu[a], Jianxun Luo[a], Zhijun Zhang[b*], Shiqi Sun [a*], Huichen Guo[a*]

[a]State Key Laboratory of Veterinary Etiological Biology, Lanzhou Veterinary Research Institute, Chinese Academy of Agricultural Sciences, Xujiaping No1, Lanzhou, Gansu, 730046, China

[b]CAS Key Laboratory of Nano-Bio Interface, Division of Nanobiomedicine, CAS Center for Excellence in Nanoscience, Suzhou Institute of Nano-Tech and Nano-Bionics, Chinese Academy of Sciences, Suzhou 215123, China

*Corresponding authors:

HC.Guo, Email: guohuichen@caas.cn, Tel: +86-931-8312213, Fax: +86-931-8340977.

SQ.Sun, E-mail: sunshiqi@caas.cn, Tel: +86-931-8312213, Fax: +86-931-8340977.

ZJ.Zhang, E-mail: zjzhang2007@sinano.ac.cn, Tel: +86-512-62872556, Fax: +86-512-62603079.

[‡]Both authors contributed equally to this work and should be considered co-first authors

Supplementary Information

Supplementary Methods

1. Stability of FeGQD@VP2 in different media

FeGQD or FeGQD@VP2 was mixed with PBS, RMPI-1640, or FBS at 1:5 ratio (v/v). The samples were set aside overnight at room temperature.

2. Hemolysis assay

Hemolysis assay was performed using fresh rat blood stabilized with EDTA. Red blood cells (RBCs) were collected by centrifugation at 3000 rpm for 3 min and then washed for three times with PBS buffer. A stock dispersion was prepared by mixing 3 mL of the centrifuged RBCs with 6 mL of PBS. Exactly 0.2 mL of the diluted RBCs was added to 0.8 mL of PBS as a negative control, to 0.8 mL of water as a positive control, and to 0.8 mL of PBS containing different concentrations of FeGQD@VP2 ranging from 10 μg mL^{-1} to 100 μg mL^{-1}. The mixtures were then incubated at 37 °C for 2 h. The percentage of hemolysis in the supernatant was measured by UV–vis analysis at 541 nm after centrifugation at 12000 rpm for 5 min.

Supplementary Figures

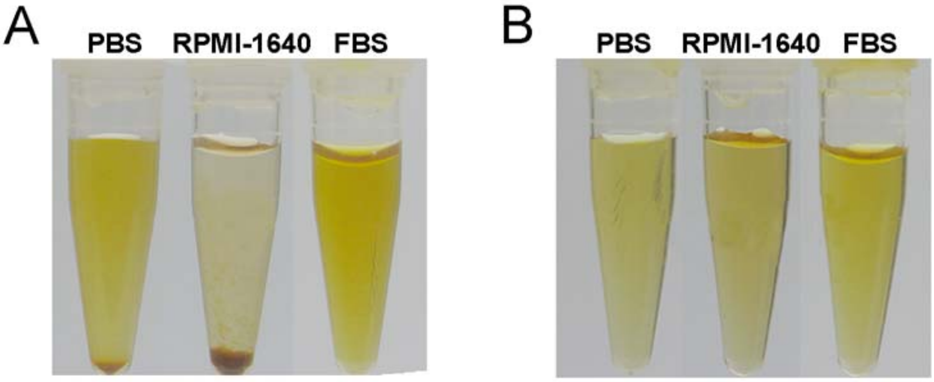

Supplementary Figure 1. Stability in different medium of FeGQD and FeGQD@VP2. (A) Stability of the FeGQD was detected in different medium as indicated, PBS (pH7.4), RPMI-1640 cultural medium and FBS. (B) Stability of the FeGQD@VP2 was detected in different medium as indicated, PBS (pH7.4), RPMI-1640 cultural medium and FBS.

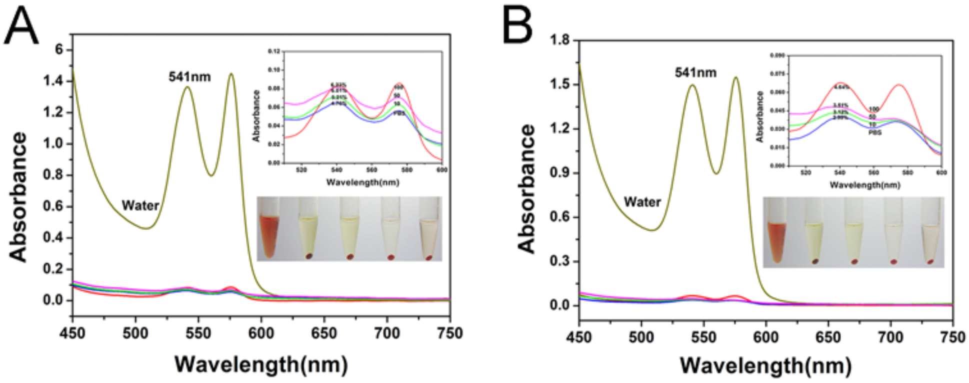

Supplementary Figure 2. Hemocompatibility of nanoparticles. (A) FeGQD and (B) FeGQD@VP2. The hemolytic activity of nanoparticles was determined by measuring the amount of hemoglobin released from RBCs, and the hemolysis percentage of nanoparticles was quantified based on the absorbance of the supernatant at 541 nm.
